# Patients' Satisfaction on Telemedicine Service in Relation to COVID-19 and Non-COVID-19 Problems: A Cross-Sectional Comparative Study

**DOI:** 10.7759/cureus.31720

**Published:** 2022-11-21

**Authors:** Manuj K Sarkar, Subhra Dey, Rajesh Kumar, Saurabh Varshney, Pratima Gupta, Boudhayan Das Munshi, Ritu LNU, Ashutosh Kumar, Vaibhav Kumar

**Affiliations:** 1 Internal Medicine, All India Institute of Medical Sciences, Deoghar, Deoghar, IND; 2 Dentistry, All India Institute of Medical Sciences, Deoghar, Deoghar, IND; 3 Otolaryngology, All India Institute of Medical Sciences, Deoghar, Deoghar, IND; 4 Microbiology, All India Institute of Medical Sciences, Deoghar, Deoghar, IND; 5 Internal Medicine, All India Institute of Medical Sciences, Kalyani, Saguna, IND; 6 Pediatrics, Lalbagh Subdivision Hospital, Murshidabad, IND; 7 Respiratory Medicine, Nalanda Medical College and Hospital, Patna, IND

**Keywords:** covid-19 pandemic, covid-19 impact of lockdown, sars-cov-2, public health problems, health care needs, tele-helpline, telemedicine (tm)

## Abstract

Background: Telemedicine service was historically started for ambulatory and hospice care patients. Since 2020, we have been in the midst of the COVID-19 pandemic. The availability of healthcare facilities became limited due to repeated locked down during the COVID-19 pandemic. Thus, telemedicine service has gained tremendous popularity among healthcare services. Telemedicine service was started at All India Institute of Medical Sciences (AIIMS), Deoghar, as a COVID Helpline facility to provide guidance and care to the home isolated COVID-19 patients during the second wave of COVID-19. But we observed that more than 40% of calls were due to non-COVID-19-related problems, but we managed the non-COVID-19-related calls by discussing with a specialist in conference calls or WhatsApp consultation. Therefore, we planned to compare individual satisfaction with telemedicine services in patients with COVID-19 and non-COVID-19-related problems.

Methods and materials: This study was a cross-sectional retrospective analysis of the register of telemedicine maintained in AIIMS, Deoghar, callers were grouped into two- COVID-19 and non-COVID-19-related problems. We obtained feedback from the patients and recorded it in a google form, collected data were analyzed in both groups. Telephonic consent was taken for participating in the study. The sample size was calculated to be 252, the COVID-19 group: 126, and the non-COVID-19 group: 126, and simple random sampling was used to choose the participants from the 730 total callers of the first month of telemedicine service. Their response was graded on 4 points Likert scale (1=Poor, 2=fair, 3=Good, 4=Excellent) and outcomes were analyzed by IBM SPSS (version 20.0) software. A p-value of <0.05 was considered statistically significant.

Objective: The primary objective is to estimate the level of satisfaction in both groups and compare their level of satisfaction. The secondary objective is to determine the department-specific telemedicine services requirement for people in need.

Results: Out of a total of 252 patients, most (54%) callers were 18-45 years old, and 44% were above 45 years old. 64% of patients were male. 90% of callers were from urban or semi-urban districts. 90% of callers had a 10th-grade or more education. 89% of patients were willing to use telemedicine services in the future. An Independent sample t-test was used to compare the means of both the groups showed a significant difference (p < 0.05) in the level of satisfaction in the COVID-19 group to the non-COVID-19 group. It showed that satisfaction in the COVID-19 group was higher than the group with non-COVID-19-related problems.

Conclusion: COVID-19 has changed the whole spectrum of healthcare needs of the community. Our study findings showed that there is a need for separate department-wise telemedicine services to provide satisfactory service for attending to problems related to that department. For example, problems with diabetes should be attended to by an endocrinologist or an internal medicine specialist. This study finding helped us to change the policy and start department-wise telemedicine service.

## Introduction

Coronavirus disease 2019 (COVID-19) caused by severe acute respiratory syndrome coronavirus 2 (SARS-CoV-2) was first detected in Wuhan city, China, in December 2019. We have witnessed many waves and variants of the virus have been detected from time to time. The pandemic has taught us many things. The world population has faced many locked down across countries from time to time, which forced us to seek telemedicine services more frequently than usual [1]. Telemedicine is defined by World Health Organization (WHO) as “The delivery of healthcare services, where distance is a critical factor, by all healthcare professionals using information and communication technologies for the exchange of valid information for the diagnosis, treatment, and prevention of disease and injuries, research and evaluation and for the continuing education of healthcare providers, all in the interests of advancing the health of individuals and their communities” [2,3]. Telemedicine service was started initially for palliative and hospice care for chronic diseases. It is required for home and community-based care for chronic disease to decrease the distance between doctors and patients and to provide day-to-day advice easily from distance without admitting the patients by the use of technology. Advancements in technology helping us to extend telemedicine services to a variety of fields including home and community-based care for chronic disease, office-based telemedicine, teleradiology, telepathology, tele-pharmacology, and hospital-based telemedicine [4]. Several studies have demonstrated that telemedicine service is comparable to a traditional hospital visit. It also reduces travel costs, reduces waiting time, and prevents unnecessary hospital visits [3]. During COVID-19, telemedicine service was used extensively for screening patients, monitoring non-serious COVID-19 patients telephonically, monitoring home-isolated patients telephonically, advising them appropriately for adequate measures based on home monitored parameters like respiratory rate, heart rate, saturation on the finger pulse oximeter, temperature on the thermometer. It was also used for many chronic diseases to monitor their disease progress, and to monitor the response after treatment. Telemedicine service was started in many countries including India to provide the best possible medical services at the time of locked down [3,5-8].

Telemedicine service was started at All India Institute of Medical Sciences (AIIMS), Deoghar, as a COVID Helpline facility to provide guidance and care to the home isolated COVID-19 patients during the second wave of COVID-19. But we observed that more than 40% of calls were due to non-COVID-19 related problems, which suggested that the community needed proper advice for health-related issues other than COVID-19. We managed the non-COVID-19-related calls by discussing with specialist department-specific consultants in conference calls or WhatsApp messaging and after getting proper advice from the consultant, we used to reply telephonically/via video call as per the requirement of the patient in need. Through the search of the literature, we could not find any published article that compared the satisfaction level of individuals who attended telemedicine services for COVID-19-related problems compared with non-COVID-19-related problems. Therefore, we planned to compare individual satisfaction with telemedicine services in patients with COVID-19 and non-COVID-19-related problems.

## Materials and methods

Study type

A cross-sectional retrospective survey study using details from the telemedicine register maintained in AIIMS, Deoghar, who sought telemedicine service during the second wave of COVID-19 in May-June 2021. The primary objective is to know patients’ satisfaction with the COVID helpline service provided by AIIMS, Deoghar, and to compare their satisfaction level who sought COVID-19-related problems vs non-COVID-19-related consultations. The secondary objective is to determine the department-specific telemedicine services requirement for people in need.

Study participants and sampling methodology

This study was done on 252 patients who used the telemedicine service of AIIMS, Deoghar in May 2021. The details of the callers were found out from the telemedicine register maintained in AIIMS, Deoghar, they were grouped into two groups, 1) those who used telemedicine service for COVID-19 related problems and 2) who used telemedicine service for non-COVID-19-related problems. We found a total of 730 callers used telemedicine service in one month. So, considering the study population as 730, we calculated the required sample size, n=252 using the formula = n/ {1+z2 p(1-p)/e2N}, COVID-19 group: 126 patients, non-COVID-19 group: 126 patients. All the callers were given serial numbers in both groups and the Simple random sampling method was used to avoid selection bias and every caller had an equal chance of selection in the study.

Confidentiality and research ethics

Collected data in google forms were recorded by independent workers not related to the investigators to reduce the risk of bias and were kept in a password-protected file and kept with the principal investigator and no individual data was shared with any other person. This study was approved by the institutional ethical committee with the approval number of IEC/AIIMS/DEO/2021-03-IND-01.

Data collection tool

We took telephonic informed consent for participating in the study and for those who were willing to take part in the study, their response was recorded in the google form. The 10-item Telemedicine Satisfaction Score (TSS) was used from a 12-item scale used in previous telehealth studies [9]. Two points namely 1) How satisfied were you with the length of time to get this appointment 2) Visual quality of the equipment was excluded as we were not giving prior appointments and we removed the question on visual quality as it depends upon the availability of the mobile network, also we did not use video consultation for all patients. Two stages of questions were set: 1) socio-demographic details and 2) a questionnaire related to the level of satisfaction (Table 1).

**Table 1:** Socio-demographic details questionnaire and questionnaire used for finding the level of satisfaction with telemedicine service

Serial no.	Socio-Demographic details questionnaire	Questionnaire related to the level of satisfaction
1	Consent for participating in the study	Voice quality of the conversation.
2	Age	Personnel comfort using the medium during the consultation.
3	Gender	Ease of getting the consultation.
4	Locality	Satisfaction with the length of consultation.
5	Residence	Satisfaction with the explanation of your treatment by the doctor.
6	Education	Skilfulness of the telemedicine team or Doctor.
7	Marital status	The courtesy, respect, sensitivity, and friendliness of the attending doctor.
8	Family status	How well your privacy was maintained?
9	Weight change during COVID-19	How well doctor answered your questions?
10	Would you like to use telemedicine services again in the future?	How satisfied you were with the treatment?

Their response was recorded on a 4 points Likert scale (1=Poor, 2=fair, 3=Good, 4=Excellent) and the total score on the 10-item TSS can range from 10 to 40, with higher scores indicating higher satisfaction.

Statistical analysis

Thus, obtained data were analyzed by Statistical Package for Social Sciences (IBM SPSS software version 20.0). Demographic data and basic characteristics of frequency distributions were compared in both groups by descriptive statistics. To compare the differences in means of responses in COVID-19 and non-COVID-19 groups, independent sample t-test analysis was done in both groups and, p < 0.05 was considered statistically significant.

## Results

**Figure 1:**
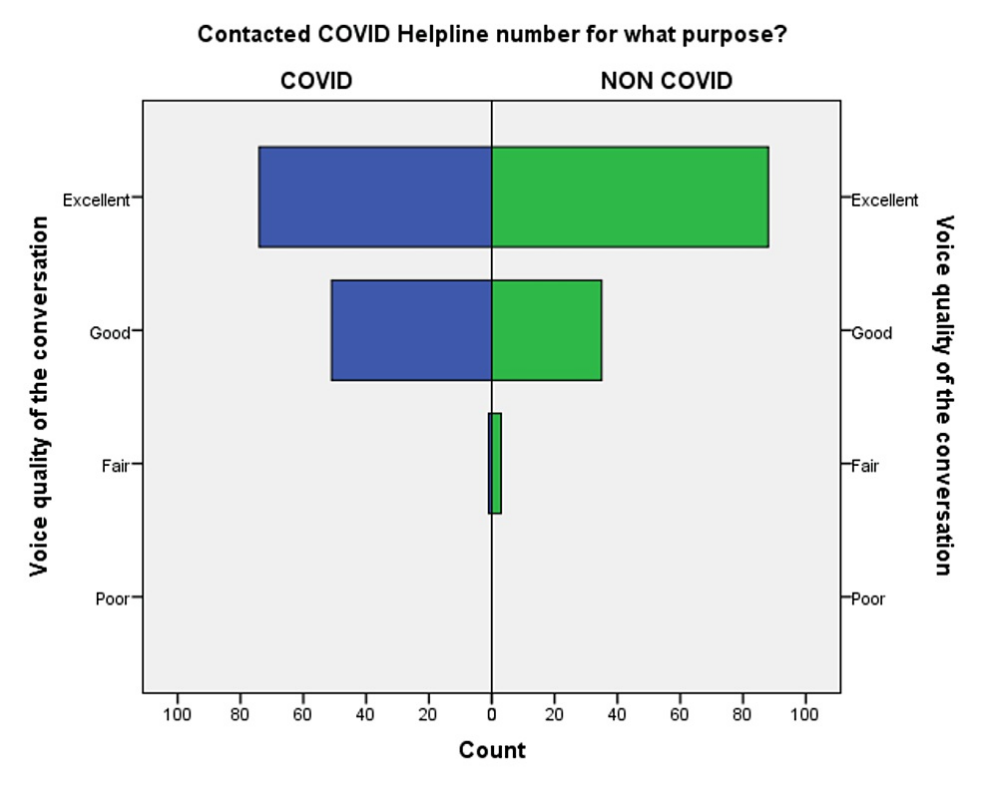
Comparison of voice quality of the conversation in the COVID-19 and non-COVID-19 groups.

**Figure 2:**
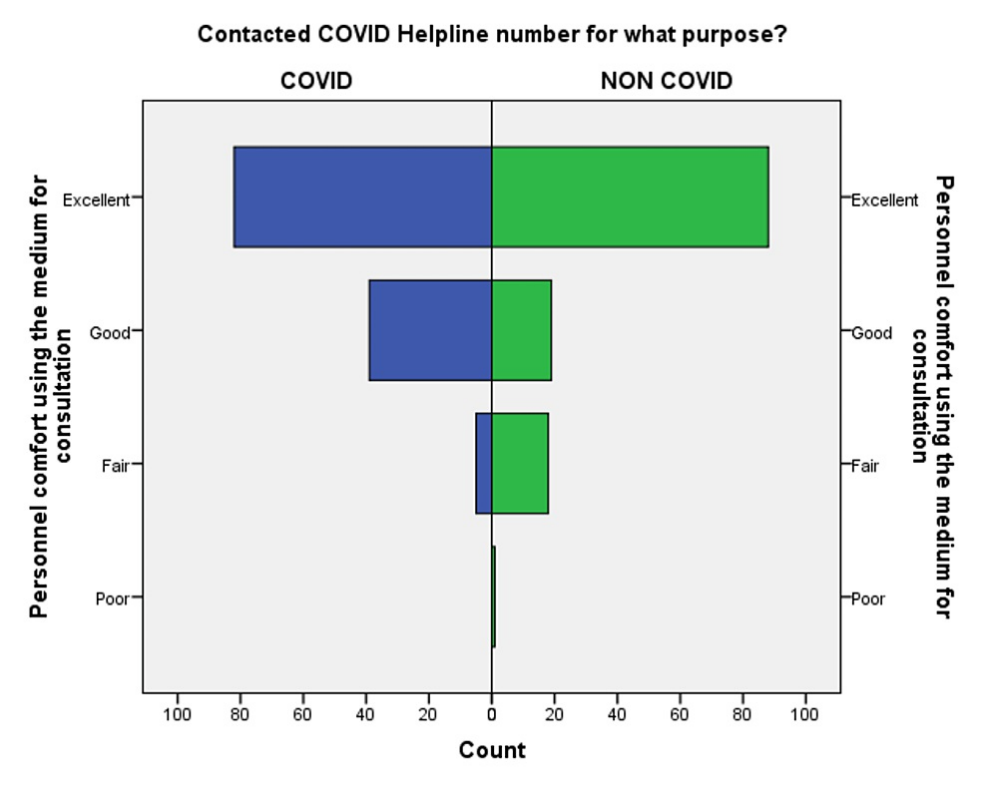
Comparison of personal comfort while using the medium during a conversation in COVID-19 and non-COVID-19 groups.

**Figure 3:**
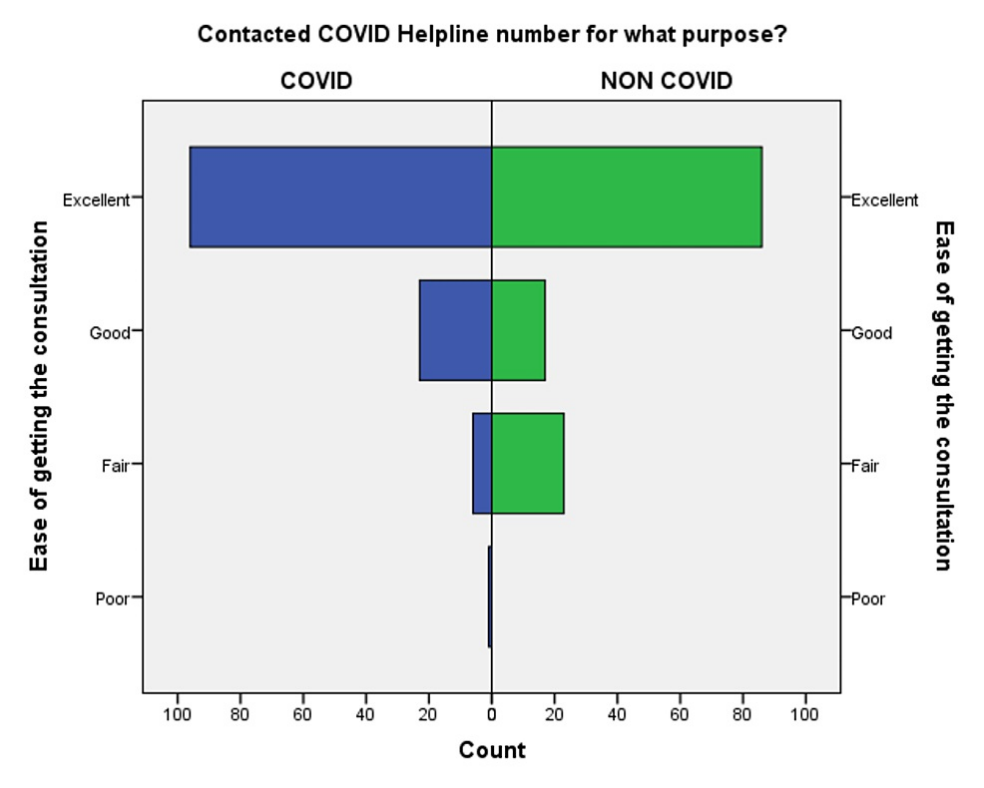
Comparison of ease of getting the consultation in COVID-19 and non-COVID-19 groups.

**Figure 4:**
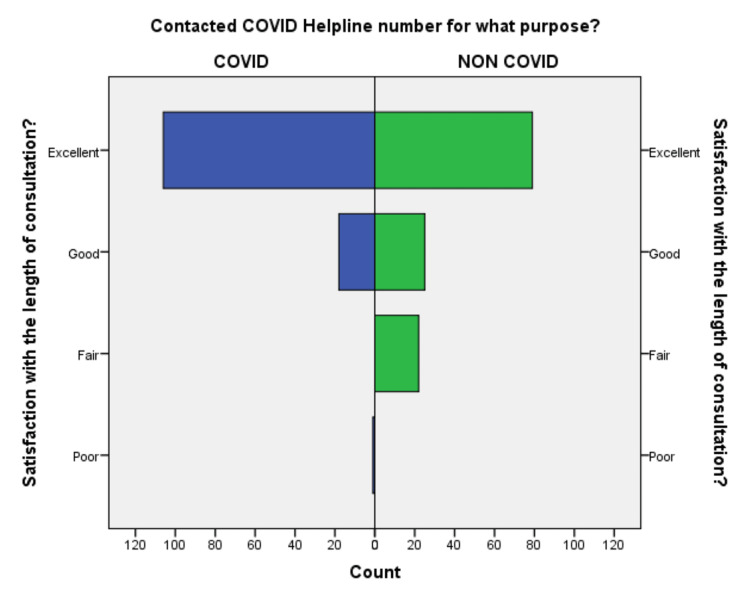
Comparison of satisfaction with the length of consultation in the COVID-19 and non-COVID-19 groups.

**Figure 5:**
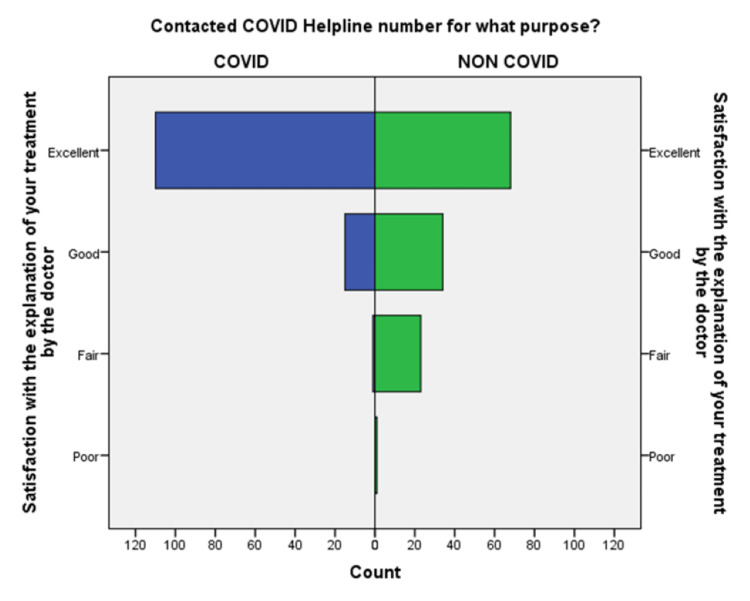
Comparison of satisfaction with an explanation of treatment by the doctor during the conversation in the COVID-19 and non-COVID-19 groups.

**Figure 6:**
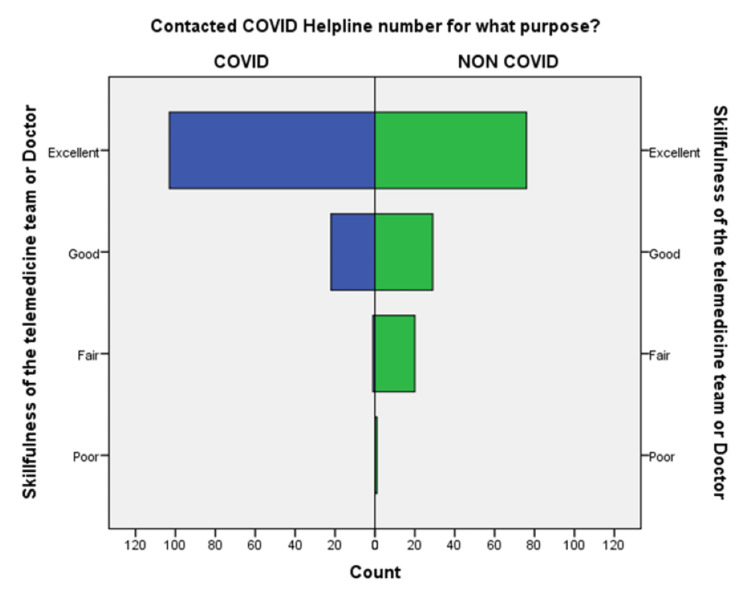
Comparison of the skillfulness of telemedicine team or doctor during a conversation in COVID-19 and non-COVID-19 groups.

**Figure 7:**
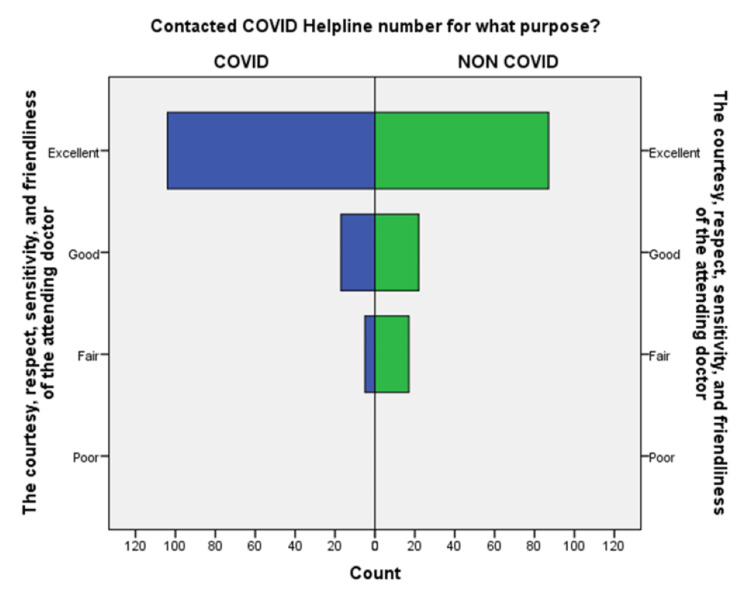
Comparison of the courtesy, respect, sensitivity, and friendliness of the doctor during the conversation in the COVID-19 and non-COVID-19 groups.

**Figure 8:**
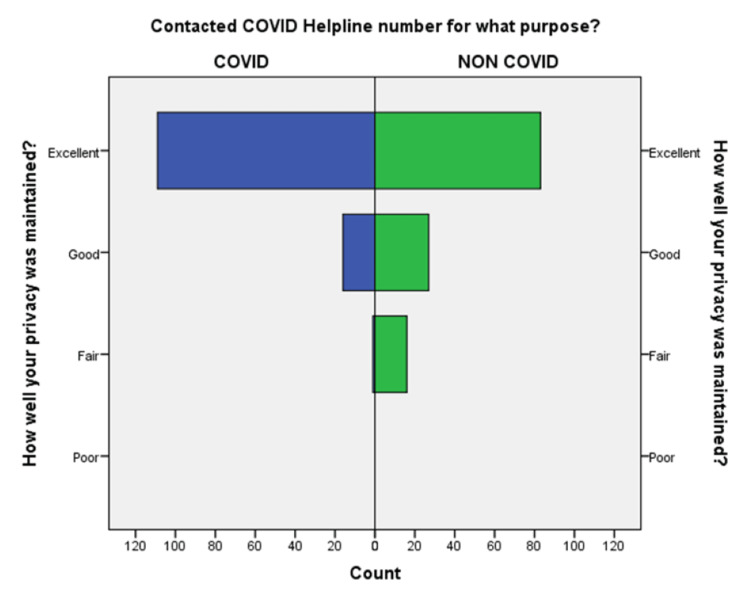
Comparison of maintenance of privacy during a conversation in the COVID-19 and non-COVID-19 groups.

**Figure 9:**
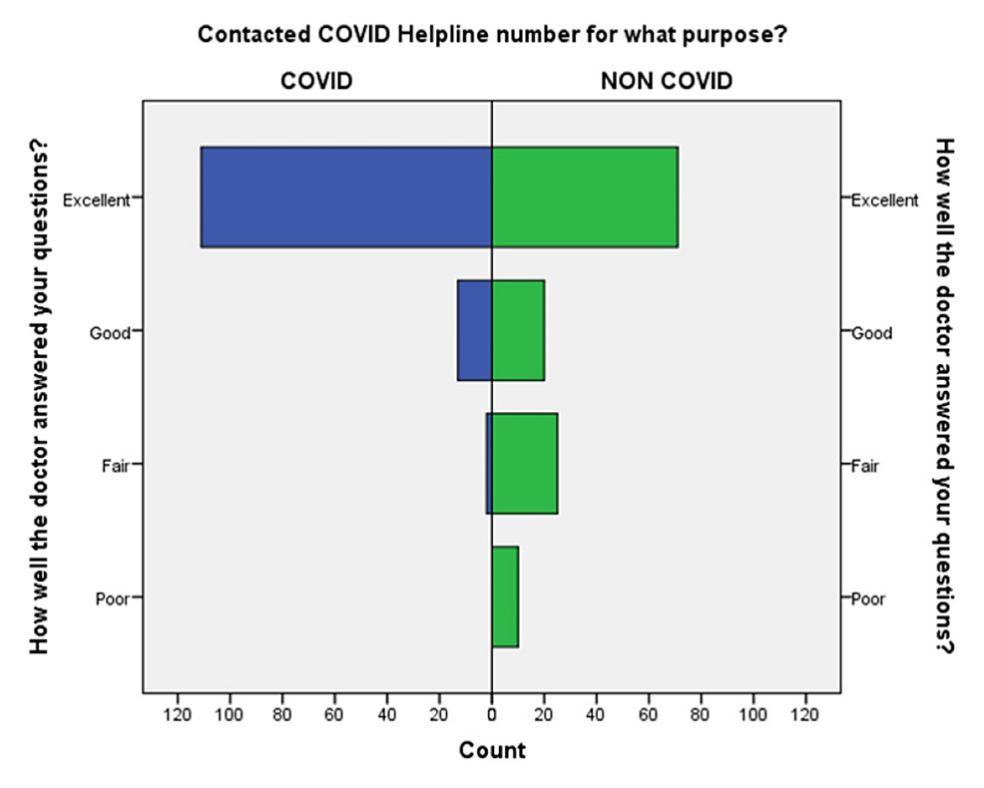
Comparison of answering wellness of doctors to questions during the conversation in COVID-19 and non-COVID-19 groups.

**Figure 10:**
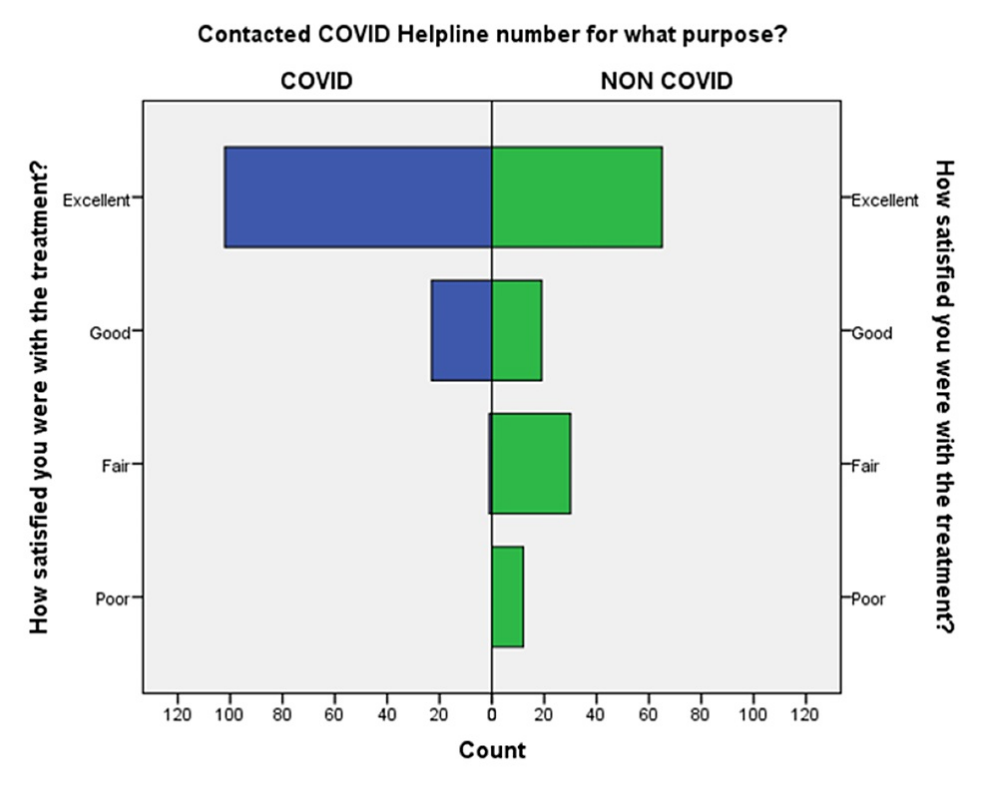
Comparison of satisfaction of patients with the treatment in the COVID-19 and non-COVID-19 groups.

Out of 252 respondents included in the study, 64% of callers were male, the majority 54% of callers were 18-45 years old, and 42% of patients were above 45 years, suggestive of adults and elderly needed healthcare facility in the community. 64% of callers were from the Deoghar district, and 36% of callers were from outside, suggestive of telemedicine could serve people who live far away from the existing healthcare facility. 62.7% of callers were from an urban area, and 27% were from a semiurban area, suggesting of the urban population was able to use the telemedicine service frequently. 90% were 10th class and above, among them, 21% were master’s degree holders, 38.5% were having bachelor’s degrees and the remaining 31% studied till 10th standard. 54.8% responded there was no change in weight, 24% lost weight and 14% gained weight during COVID-19. This suggests that there is a need for an awareness program for rural and uneducated people to be able to use telemedicine services. The differences in the responses to the questionnaire regarding the level of satisfaction are compared in Figures 1-10.

An independent sample t-test was used to compare the means of both the groups showed a significant difference (p < 0.05) in the level of satisfaction in all the later eight questions except the initial two. This finding suggests that the people in the COVID-19 group were significantly more satisfied than the non-COVID-19 group (Table 2). This also shows that satisfaction with the treatment had the maximum differences in the mean level of satisfaction in the COVID-19 group compared with the non-COVID-19 group. We also found that the questions where professional expertise is required like: (a) How well doctors answered your question, (b) satisfaction with the explanation of your treatment by the doctor, (c) skillfulness of the telemedicine team or doctor, showed maximum differences in the mean level of satisfaction. These findings are suggestive of the presence of subject experts, proper training of the telemedicine team, and calls attending doctors can improve telemedicine service and help us to provide satisfactory telemedicine service. Department-specific telemedicine is an essential component of telemedicine. The subject experts should attend the calls, rather than calls attended by junior resident doctors or any less experienced doctor.

**Table 2:** Group statistics and independent sample t-test based p-value (sample size in each group, n=126) in COVID-19 and non-COVID-19 group.

Serial Number	Questionnaire related to the level of satisfaction	Purpose of contacting telemedicine	Mean	Standard Deviation	P-value
1	Voice quality of the conversation	COVID-19	3.58	0.512	0.144
		non-COVID-19	3.67	0.519	
2	Personnel comfort using the medium during the consultation	COVID-19	3.61	0.565	0.4
		non-COVID-19	3.54	0.766	
3	Ease of getting the consultation	COVID-19	3.7	0.597	0.025
		non-COVID-19	3.5	0.787	
4	Satisfaction with the length of consultation	COVID-19	3.83	0.435	<0.001
		non-COVID-19	3.45	0.776	
5	Satisfaction with the explanation of your treatment by the doctor	COVID-19	3.87	0.366	<0.001
		non-COVID-19	3.34	0.802	
6	Skilfulness of the telemedicine team or Doctor	COVID-19	3.81	0.414	<0.001
		non-COVID-19	3.43	0.784	
7	The courtesy, respect, sensitivity, and friendliness of the attending doctor	COVID-19	3.79	0.5	<0.004
		non-COVID-19	3.56	0.722	
8	How well your privacy was maintained?	COVID-19	3.86	0.373	<0.001
		non-COVID-19	3.53	0.712	
9	How well doctor answered your questions?	COVID-19	3.87	0.387	<0.001
		non-COVID-19	3.21	1.022	
10	How satisfied you were with the treatment?	COVID-19	3.8	0.42	<0.001
		non-COVID-19	3.09	1.066	
11	Would you like to use telemedicine services again in the future?	COVID-19	1.05	0.306	<0.001
		non-COVID-19	1.33	0.702	

**Figure 11:**
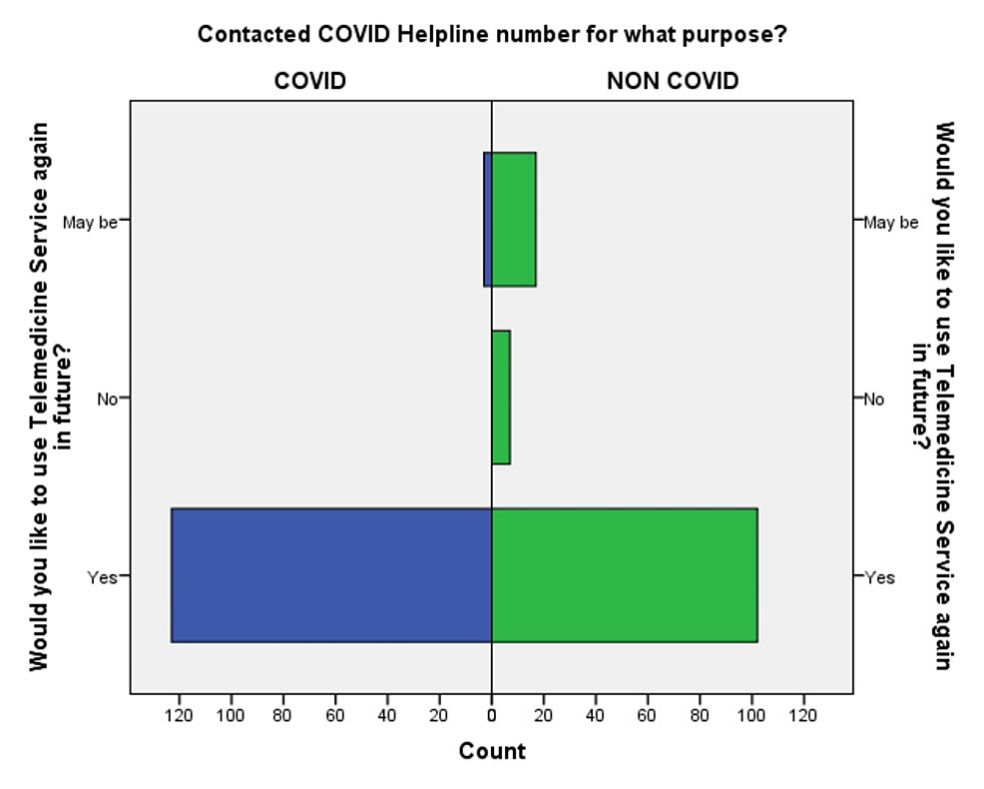
Willingness in the future use of telemedicine service in all the participants in both groups.

Factor analysis in nonparametric (Spearman’s correlation) regression showed that the responses to the questionnaires regarding the level of satisfaction were linearly and positively correlated with one another suggestive of individuals who were satisfied, were satisfied with all parameters and vice-versa (Table 3). Moreover, the participants in the COVID-19 group were willing to use telemedicine service again in the future more compared to the non-COVID-19 groups and the mean difference in willingness to use again in the future was statistically significant (Table 2, Figure 11).

**Table 3:** Nonparametric correlation (Spearman’s correlation)

			Ease of getting the consultation	Satisfaction with the length of consultation?	Satisfaction with the explanation of your treatment by the doctor	Voice quality of the conversation	Skillfulness of the telemedicine team or Doctor	The courtesy, respect, sensitivity, and friendliness of the attending doctor	How well your privacy was maintained?	How satisfied you were with the treatment?	Personnel comfort using the medium for consultation	How well the doctor answered your questions?	Would you like to use Telemedicine Service again in future?
Spearman's rho	Ease of getting the consultation	Correlation Coefficient	1.000	.713^**^	.511^**^	.424^**^	.595^**^	.778^**^	.779^**^	.613^**^	.621^**^	.566^**^	-.336^**^
		Sig. (2-tailed)		.000	.000	.000	.000	.000	.000	.000	.000	.000	.000
		N	252	251	252	252	252	252	252	252	252	252	252
	Satisfaction with the length of consultation?	Correlation Coefficient	.713^**^	1.000	.634^**^	.376^**^	.713^**^	.694^**^	.724^**^	.692^**^	.545^**^	.694^**^	-.377^**^
		Sig. (2-tailed)	.000		.000	.000	.000	.000	.000	.000	.000	.000	.000
		N	251	251	251	251	251	251	251	251	251	251	251
	Satisfaction with the explanation of your treatment by the doctor	Correlation Coefficient	.511^**^	.634^**^	1.000	.229^**^	.630^**^	.513^**^	.539^**^	.728^**^	.342^**^	.744^**^	-.455^**^
		Sig. (2-tailed)	.000	.000		.000	.000	.000	.000	.000	.000	.000	.000
		N	252	251	252	252	252	252	252	252	252	252	252
	Voice quality of the conversation	Correlation Coefficient	.424^**^	.376^**^	.229^**^	1.000	.350^**^	.397^**^	.429^**^	.330^**^	.607^**^	.335^**^	-.207^**^
		Sig. (2-tailed)	.000	.000	.000		.000	.000	.000	.000	.000	.000	.001
		N	252	251	252	252	252	252	252	252	252	252	252
	Skillfulness of the telemedicine team or Doctor	Correlation Coefficient	.595^**^	.713^**^	.630^**^	.350^**^	1.000	.695^**^	.725^**^	.722^**^	.554^**^	.705^**^	-.480^**^
		Sig. (2-tailed)	.000	.000	.000	.000		.000	.000	.000	.000	.000	.000
		N	252	251	252	252	252	252	252	252	252	252	252
	The courtesy, respect, sensitivity, and friendliness of the attending doctor	Correlation Coefficient	.778^**^	.694^**^	.513^**^	.397^**^	.695^**^	1.000	.848^**^	.691^**^	.647^**^	.604^**^	-.425^**^
		Sig. (2-tailed)	.000	.000	.000	.000	.000		.000	.000	.000	.000	.000
		N	252	251	252	252	252	252	252	252	252	252	252
	How well your privacy was maintained?	Correlation Coefficient	.779^**^	.724^**^	.539^**^	.429^**^	.725^**^	.848^**^	1.000	.732^**^	.705^**^	.652^**^	-.419^**^
		Sig. (2-tailed)	.000	.000	.000	.000	.000	.000		.000	.000	.000	.000
		N	252	251	252	252	252	252	252	252	252	252	252
	How satisfied you were with the treatment?	Correlation Coefficient	.613^**^	.692^**^	.728^**^	.330^**^	.722^**^	.691^**^	.732^**^	1.000	.505^**^	.764^**^	-.476^**^
		Sig. (2-tailed)	.000	.000	.000	.000	.000	.000	.000		.000	.000	.000
		N	252	251	252	252	252	252	252	252	252	252	252
	Personnel comfort using the medium for consultation	Correlation Coefficient	.621^**^	.545^**^	.342^**^	.607^**^	.554^**^	.647^**^	.705^**^	.505^**^	1.000	.449^**^	-.334^**^
		Sig. (2-tailed)	.000	.000	.000	.000	.000	.000	.000	.000		.000	.000
		N	252	251	252	252	252	252	252	252	252	252	252
	How well the doctor answered your questions?	Correlation Coefficient	.566^**^	.694^**^	.744^**^	.335^**^	.705^**^	.604^**^	.652^**^	.764^**^	.449^**^	1.000	-.481^**^
		Sig. (2-tailed)	.000	.000	.000	.000	.000	.000	.000	.000	.000		.000
		N	252	251	252	252	252	252	252	252	252	252	252
	Would you like to use Telemedicine Service again in future?	Correlation Coefficient	-.336^**^	-.377^**^	-.455^**^	-.207^**^	-.480^**^	-.425^**^	-.419^**^	-.476^**^	-.334^**^	-.481^**^	1.000
		Sig. (2-tailed)	.000	.000	.000	.001	.000	.000	.000	.000	.000	.000	
		N	252	251	252	252	252	252	252	252	252	252	252
**. Correlation is significant at the 0.01 level (2-tailed).													

## Discussion

Our study was done to determine the level of satisfaction of the people who called our facility for various health-related issues. Though we started a Tele helpline service in AIIMS, Deoghar, for home-isolated COVID-19-positive patients during the second wave of COVID-19. We have seen that more than 40% of callers were for non-COVID-19-related problems. This finding suggested that there is an emergency need for healthcare services in the community mainly for those with chronic diseases, who have not been able to get an appointment due to the decrease in ambulatory services secondary to COVID-19-related restrictions.

Telemedicine is a very essential component of the healthcare system. Agarwal et al. noted that 75% of physicians are concentrated in cities but 69% of the general population lives in rural areas. This means only 25% of doctors are accessible to 69% of the population; therefore, telemedicine can serve to remove the distance factor, and thereby it can allow the general population including rural people to avail healthcare facilities even staying in villages [10]. In our study, we have seen that 90% population who used telemedicine services were from urban or semi-urban areas, this means that majority of the population who lives in rural areas are not using telemedicine services adequately. we must initiate an awareness campaign to raise awareness regarding telemedicine services available in our country.

Telemedicine service has evolved since its inception. Initially, it was started for palliative and hospice care, it has evolved in many directions over the past few decades with the use of wireless service, cell phones, the internet, video calling, photo sharing, etc. The government of India has taken many steps to implement telemedicine service and during COVID-19, it has gained popularity tremendously [3,10-12].

Robinson et al. studied the issues of rural palliative care and they found that rural people are vulnerable to inadequate healthcare services and a tailored approach is required in rural populations [13]. We also found that rural populations are vulnerable to inadequate utilization of telemedicine services despite its availability with a single dial by a cell phone. Nesbitt et al. studied the status of perceptions of health care quality in rural communities with telemedicine and found that telemedicine introduction into rural communities will be associated with increased acceptance by local communities and will decrease the travel by rural populations in seeking health care access [14]. Several studies have demonstrated that live video conferencing with patients can alleviate physical contact, thereby reducing the risk of transmission of communicable diseases [15].

Telemedicine practice guidelines, as proposed by the national medical commission in India emphasizes that doctors or registered medical practitioners should receive training in telemedicine that includes an understanding of the limitation of telemedicine [16]. In our institute, we trained our telemedicine consultation team regarding the management of COVID-19-related problems, therefore satisfaction in the COVID-19 group was higher than the non-COVID-19 group. This suggests that satisfactory service to patients depends upon the presence of experts or the trained person who would know how to refer the patient to the right clinic or physician for teleconsultation.

## Conclusions

Our study showed that patient satisfaction was higher when dealing with COVID-19-related questions than the non-COVID-19-related issues. This suggests that proper planning and training of the telemedicine team is very much essential for providing proper and satisfactory telemedicine service. In our study, telemedicine service in our institute was intended for providing service to patients in home isolation during the second wave of COVID-19 in India, and our team was trained in managing the need of these patients. This finding suggests that for providing satisfactory telemedicine service, training for managing non-COVID-19-related issues is very essential. Though there was a difference in the level of satisfaction, overall, 89% of patients were willing to use telemedicine service in the future suggesting that telemedicine as a tool for health care delivery works very well and that we should encourage all to use telemedicine service whenever feasible. We also found that rural and people with little education used telemedicine services less than 10%. This suggests that an awareness campaign for use of telemedicine can prove pivotal for the future expansion of telemedicine services in our society.
